# Finite element analysis of the effects of different ossification types and spinal canal occupancy rates on dynamic spinal cord stress in cervical ossification of the posterior longitudinal ligament

**DOI:** 10.3389/fbioe.2026.1832964

**Published:** 2026-07-21

**Authors:** Xiao Zhang, Hongyang Zhao, Yulong Zhao, Wenbo Gu, Yu Yang, Donghui Cao, Xi Zhu, Xusheng Li, Haifeng Yuan

**Affiliations:** 1 General Hospital of Ningxia Medical University, Yinchuan, Ningxia, China; 2 Ningxia Medical University, Yinchuan, Ningxia, China; 3 Qinghai University Affiliated Hospital, Xining, Qinghai, China

**Keywords:** biomechanics, cervical ossification of the posterior longitudinal ligament, finite element analysis, spinal canal occupancy rate, spinal cord stress

## Abstract

**Objective:**

To investigate the effects of ossification type and spinal canal occupancy rate on dynamic spinal cord stress in cervical ossification of the posterior longitudinal ligament (C-OPLL) using finite element analysis.

**Methods:**

A validated C2-C7 finite element model was constructed from CT and MRI data of a healthy volunteer. Three C-OPLL types (focal, segmental, continuous) were simulated at 20%, 40%, and 60% occupancy rates. Spinal cord stress was analyzed under nine flexion-extension angles (+20° to −20°).

**Results:**

Spinal cord stress increased exponentially at 60% occupancy across all types. Focal ossification produced the highest stress (0.1196 MPa at 60% occupancy/maximum flexion), 30.4% higher than continuous type. At 60% occupancy, the stress sensitivity threshold decreased to ±5° motion. Dural stress consistently exceeded spinal cord parenchyma stress (1.6–2.1 times).

**Conclusion:**

In this single-subject finite element model at C4-C5, focal C-OPLL produced the highest peak spinal cord stress at this level. The 60% occupancy rate was identified as a critical biomechanical threshold above which stress increased exponentially and became highly sensitive to minor motion (±5°). These findings are hypothesis-generating and suggest that patients with ≥60% occupancy at C4-C5 may warrant close follow-up. However, because this study is based on a single healthy volunteer, the proposed thresholds require validation through patient-specific models and prospective studies before clinical application.

## 1 Introduction

Ossification of the Posterior Longitudinal Ligament of the Cervical Spine (C-OPLL) is one of the most common degenerative cervical spine diseases in East Asian populations, characterized by ectopic ossification of the posterior longitudinal ligament leading to spinal canal stenosis and spinal cord compression, subsequently causing severe neurological symptoms such as sensorimotor dysfunction of the extremities and bladder/bowel dysfunction (Inamasu et al., 2006). Epidemiological studies have shown that the prevalence of C-OPLL in the Chinese population is approximately 0.077‰, predominantly affecting middle-aged and elderly individuals over 40 years old, with a male-to-female ratio of approximately 2:1 (Cui et al., 2025; Liang et al., 2019). Although the pathological mechanism of C-OPLL has not been fully elucidated, the interaction between genetic susceptibility factors (such as COL6A1, IL17RC gene polymorphisms) and metabolic factors (diabetes, obesity) has been confirmed to participate in its pathogenesis (Kong et al., 2007; Singh et al., 2024; Wang et al., 2018).

According to the imaging distribution characteristics of ossification, Tsuyama et al. classified C-OPLL into four types: focal, segmental, continuous, and mixed (Tsuyama, 1984). Among these, central plateau-type ossification, due to its direct contact with the ventral spinal cord, is considered the most clinically significant pathological type. The spinal canal occupancy rate, an important indicator for quantifying the degree of ossification, is typically defined as the ratio of the maximum thickness of the ossification to the anteroposterior diameter of the spinal canal. Previous studies have shown that when the occupancy rate exceeds 60%, the incidence of myelopathy increases significantly (Kwok and Cheung, 2020). However, the degree of static compression is not always positively correlated with the severity of clinical symptoms; some patients with mild compression may present with severe neurological dysfunction, while some patients with severe compression may remain asymptomatic for long periods (Chang et al., 2010). This phenomenon suggests that in addition to static anatomical stenosis, dynamic cervical spine motion may play a key role in the mechanism of spinal cord injury.

Cervical Range of Motion (C-ROM) is an important indicator for assessing cervical spine functional status. The normal sagittal range of motion in adults is approximately 40°–45° for flexion and 80° for extension (Teare-Ketter et al., 2021). In patients with C-OPLL, paravertebral muscle spasm, abnormal ligament tension, and pain protection mechanisms often lead to restricted C-ROM. However, even within the restricted range of motion, cervical flexion-extension movements can significantly affect spinal cord stress distribution by altering the relative position of the spinal cord and ossification, regulating cerebrospinal fluid hydrodynamics, and changing dentate ligament tension (Ito et al., 2015b). Finite element studies by Nishida et al. confirmed that static and dynamic compression factors can synergistically lead to increased spinal cord stress, but their study was limited to a single ossification type and a relatively small range of flexion-extension angles (Nishida et al., 2015a). Therefore, conducting systematic finite element studies covering multiple ossification types, varying degrees of spinal canal occupancy, and the full range of flexion-extension motion is of great theoretical value and clinical significance for comprehensively revealing the biomechanical mechanisms of spinal cord injury in C-OPLL and filling the gaps in existing research.

Finite Element Analysis (FEA), as a non-invasive biomechanical research tool, can quantitatively analyze the stress-strain distribution characteristics of complex anatomical structures under precisely controlled boundary conditions (Davies et al., 2024). In recent years, FEA has been increasingly widely used in spinal cord biomechanics research, providing an important tool for understanding the pathological mechanisms of C-OPLL and optimizing surgical strategies (Xue H. et al., 2023). However, existing studies mostly focus on a single ossification type or fixed cervical spine positions, lacking systematic comparative studies of different types of C-OPLL under varying degrees of spinal canal occupancy and the full range of flexion-extension motion. Therefore, this study constructed a three-dimensional finite element model incorporating a detailed spinal cord complex structure. By simulating the biomechanical behavior of continuous, focal, and segmental C-OPLL at 20%, 40%, and 60% spinal canal occupancy rates and within ±20° flexion-extension range, this study aimed to: (1) clarify the differential effects of different ossification types on spinal cord stress distribution; (2) determine the dose-response relationship between spinal canal occupancy rate and spinal cord stress sensitivity; (3) identify critical cervical motion angles leading to significant increases in spinal cord stress; and (4) provide biomechanical evidence for individualized treatment and activity restrictions in patients with C-OPLL.

## 2 Materials and methods

### 2.1 Study subject and imaging data

This study was approved by the Ethics Committee (approval No. KYLL-2025–01019). A healthy adult male volunteer was recruited. The volunteer had no history of cervical spine trauma, surgery, tumor, infection, or congenital malformation. Thin-slice scanning of the C2-C7 segments was performed using a Philips Brilliance 16-slice CT. Simultaneously, T1 and T2-weighted images were obtained using 1.5T MRI to clearly display soft tissue anatomical structures such as the spinal cord, dura mater, and cerebrospinal fluid. Measured by the Borden method, the volunteer’s cervical physiological curvature arc-chord distance was 8.60°mm, indicating normal cervical curvature (Oh et al., 2021).

### 2.2 Finite element model construction

**FIGURE 1:**
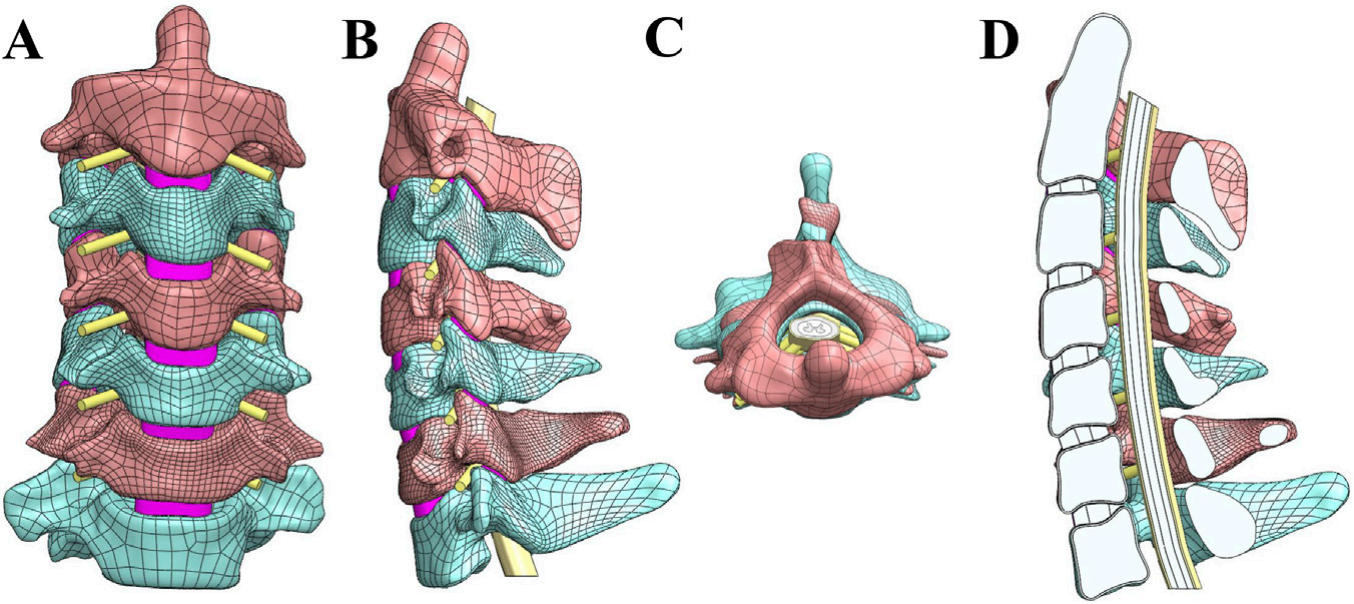
Multi-view display of the three-dimensional finite element model of the cervical spine. **(A)** Posteroanterior view; **(B)** Lateral view; **(C)** Transverse view; **(D)** Sagittal sectional view.

CT data in DICOM format were imported into Mimics 21.0 software. Based on the grayscale threshold method, the bony contours of the C2-C7 vertebral bodies were extracted, and isolated pixels were removed using the region-growing algorithm. The generated STL files were imported into Geomagic Wrap 2021 software for surface smoothing, hole filling, and polygon optimization, ultimately constructing high-precision Non-Uniform Rational B-Spline surface models. Cortical and cancellous bone models were constructed separately using the overall offset method (offset set at 0.4 mm) to accurately simulate vertebral anatomy (Yu et al., 2020). Component assembly was completed in Solidworks 2017 software. Based on anatomical parameters, an intervertebral disc complex including the annulus fibrosus, nucleus pulposus, endplates, and facet joint cartilage was constructed. Based on MRI images and literature data, a spinal cord complex model including gray matter, white matter, cerebrospinal fluid layer, and dura mater was reconstructed (Khadka et al., 2020) (Figure 1).

### 2.3 Material properties and contact definitions

All model components were assigned corresponding material properties (detailed in Table 1) (Bevill et al., 2007; Boruah et al., 2017; Huang et al., 2018; Ichihara et al., 2001; Nishida et al., 2014; Nishida et al., 2015b; Persson et al., 2010; Stapleton et al., 2011). Among these, gray matter and white matter were defined as hyperelastic materials; cerebrospinal fluid was simulated as Eulerian elements, defined as a Newtonian fluid (viscosity 1 mPas); the dura mater, cortical bone, cancellous bone, and intervertebral discs were defined as linear elastic materials. In Abaqus 2021 software, interactions between components were defined: contact between the spinal cord and ossification/vertebral bodies was set as frictionless; the Eulerian-Lagrangian coupling algorithm was used between cerebrospinal fluid and the spinal cord/dura mater to simulate fluid-structure interaction effects.

**TABLE 1:** Material properties and element types used in this study model.

Component	Material behavior	Material properties	References
Cortical bone	Linear elastic	E = 12000 MPa, ν = 0.29, ρ = 1.83E-09 tonne/mm^3^	Boruah et al. (2017)
Cancellous bone	Linear elastic	E = 100 MPa, ν = 0.29, ρ = 1.00E-09 tonne/mm^3^	Bevill et al. (2007)
Ossification	Rigid body	ρ = 1.83E-09 tonne/mm^3^	Nishida et al. (2014)
Articular cartilage	Linear elastic	E = 10 MPa, ν = 0.30, ρ = 1.20E-09 tonne/mm^3^	Huang et al. (2018)
Spinal dura mater	Linear elastic	E = 80 MPa, ν = 0.49, ρ = 1.174E-09 tonne/mm^3^	Persson et al. (2010)
Cerebrospinal fluid	Newtonian fluid	ρ = 1000 kg/m^3^, η = 1 MPa s	Persson et al. (2010)
White matter	Hyperelastic	μ = 4.0 kPa, α = 12.5	Ichihara et al. (2001)
Gray matter	Hyperelastic	μ = 4.1 kPa, α = 14.7	Ichihara et al. (2001)
Extradural nerve root	Linear elastic	E = 80 MPa, ν = 0.49	Nishida et al. (2015b)
Intradural nerve root	Nonlinear elastic-plastic	Stress-strain curve	Stapleton et al. (2011)
Denticulate ligament	Nonlinear elastic-plastic	Stress-strain curve	Stapleton et al. (2011)

E – elastic modulus; ν – Poisson’s ratio; η – viscosity; ρ – density; μ – shear modulus; α – strain hardening exponent.

### 2.4 Mesh generation and convergence analysis

A hybrid meshing strategy was adopted. Key regions such as the spinal cord, dura mater, and cerebrospinal fluid were locally refined using high-precision hexahedral elements, with a final mesh size of 0.2 mm (Tadepalli et al., 2011). Cortical bone, cancellous bone, and intervertebral discs were meshed with tetrahedral elements at a size of 1 mm. Mesh convergence testing confirmed that when the mesh size was reduced by 50%, the change in maximum von Mises stress of the spinal cord was less than 5%, indicating that the current mesh density achieves a good balance between computational accuracy and efficiency (Ayturk and Puttlitz, 2011). The final model contained a total of 1,751,548 nodes and 896,547 elements (Supplementary Figure S1).

### 2.5 Boundary conditions and loading

In the analysis, all degrees of freedom of the inferior surface of the C7 vertebra, the caudal end of the spinal cord, and the distal ends of bilateral nerve roots were fixed. The superior surface of the C2 vertebra and the cranial end of the spinal cord were connected through kinematic coupling constraints (Kumar and Kumar, 2023). Rotational displacement was applied to the C2 vertebra to simulate sagittal flexion-extension motion (20° extension to 20° flexion, 5° increments). The quasi-static assumption was adopted because physiological cervical motion occurs at low velocities (≈10°–30°/s) where inertial effects are negligible (Equation 1) (Khuyagbaatar et al., 2015; Li and Dai, 2009). No failure criterion was embedded, as the objective was comparative biomechanics rather than absolute injury prediction. CSF was modeled as a Newtonian fluid using the coupled Eulerian-Lagrangian method (Eulerian mesh 0.3°mm, initial CSF volume fraction 0 within cord, one elsewhere; pressure followed linear Us-Up EOS with bulk modulus 2.2 GPa) (Equation 4). The hyperelastic tissue behavior was described by Equation 2, while the linear elastic response of bony structures followed Equation 3; contact pressure was governed by Equation 5. The governing equations are as follows:

∇·σ+f_b=0 (1)

$${W=C}_{1}\left(I_{1}-3\right)+C_{2}\left(I_{2}-3\right)+\left(1/2\right)K{\left(J-1\right)}^{2}$$ (2)

σ=D·ε (3)

$$\tau =\mu \cdot \dot{\gamma }$$ (4)

p∝1/R (5)

where σ is the Cauchy stress tensor, f_b is body force, W is strain energy, I_1_, I_2_ are deviatoric invariants, J is volume ratio, K is bulk modulus, D is elastic stiffness, ε is strain, τ is shear stress, μ is dynamic viscosity, γ̇ is shear rate, p is contact pressure, and R is radius of curvature.

### 2.6 Model validation

To verify the biomechanical reliability of the model, two independent validation experiments were performed (Supplementary Figure S2): (1) Kinematic validation: Simulating cervical spine ±20° flexion-extension motion, the three-dimensional spatial displacements of the spinal cord at the C3-C7 levels were calculated and compared with *in vivo* MRI measurements reported by Stoner et al. (2019). (2) Mechanical validation: Simulating spinal cord vertical compression tests, a concentrated load of 0.8 N was applied to the mid-segment of the spinal cord, and the force-displacement curve was recorded and compared with *in vitro* experimental data published by Hung et al. and Hung et al. (1982), Xue F. et al. (2023).

### 2.7 C-OPLL model construction and condition design

**FIGURE 2:**
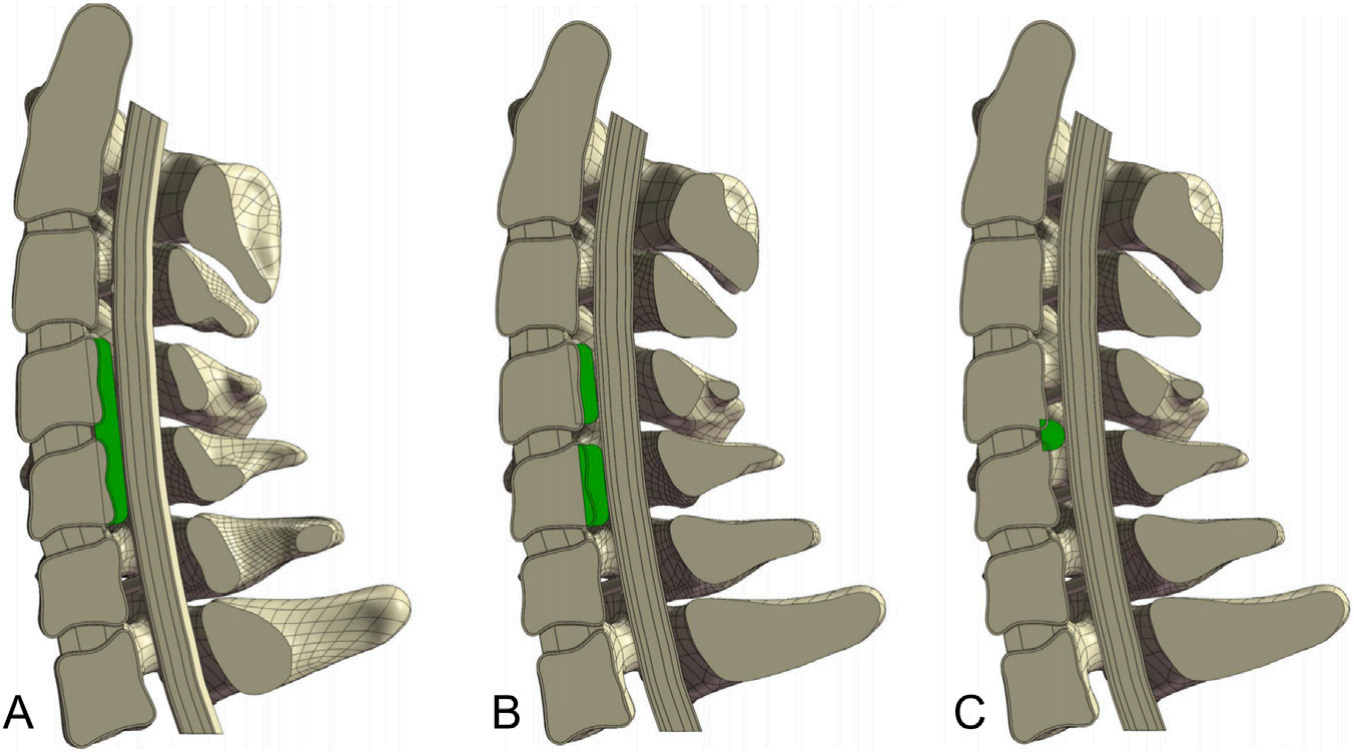
Three C-OPLL models. **(A)** Continuous type; **(B)** Segmental type; **(C)** Focal type.

Based on the validated normal cervical spine model, three clinically common central plateau-type posterior longitudinal ligament ossification models were constructed at the posterior edge of the C4-C5 vertebral bodies (Figure 2) (Tsuyama, 1984): (1) Focal type: ossification localized, mainly concentrated at the C4-C5 intervertebral disc level; (2) Segmental type: ossification located at the posterior edges of the C4 and C5 vertebral bodies respectively, discontinuous between segments; (3) Continuous type: ossification cord-like, longitudinally spanning C4-C5. All ossification models were simplified as rigid bodies to simulate their compressive effect on the spinal cord. Given that the elastic modulus of mature OPLL (≈10 GPa) is several orders of magnitude higher than that of the spinal cord (≈0.01 MPa), with a stiffness ratio exceeding 10^5^, the ossification was simplified as a rigid body. This assumption is widely adopted in prior finite element studies of compressive spinal cord injury and has negligible impact on the predicted spinal cord stress distribution trends (Khuyagbaatar et al., 2018). A full factorial design was adopted to systematically evaluate the effects of three key factors: (1) Ossification type: focal, segmental, continuous; (2) Spinal canal occupancy rate: defined as the ratio of the maximum anteroposterior diameter of the ossification to the anteroposterior diameter of the corresponding spinal canal segment, set at 20%, 40%, and 60% respectively to simulate mild, moderate, and severe compression; (3) Cervical flexion-extension angle: as described above, nine different sagittal angles. Through these combinations, a total of 81 independent analysis conditions were generated. In each condition, the ossification was first translated to the predetermined occupancy rate position to generate initial compression, then kept at a fixed relative position with the vertebral bodies while simulating cervical flexion-extension motion. The peak von Mises stress distributions in the dura mater, gray matter, and white matter of the compressed spinal cord segment were recorded and analyzed for each condition.

## 3 Results

### 3.1 Model validation

In the cervical flexion-extension validation experiment, the anteroposterior displacement range of the C3-C7 spinal cord predicted by this model was 0.8–2.3 mm, which was highly consistent with the *in vivo* MRI measurement data (0.6–2.1 mm), with an average error of less than 15%. In the vertical compression validation, the spinal cord force-displacement curve exhibited a typical nonlinear “J-shaped” characteristic, consistent with the *in vitro* experimental results; the initial stiffness was 0.12 N/mm, within the 0.10–0.15 N/mm range reported in the literature. These validation results confirmed that this model possesses good predictive accuracy and can be used for subsequent C-OPLL simulation analysis (Supplementary Figure S3).

### 3.2 Stress distribution characteristics of spinal cord and dura mater in different ossification types

**FIGURE 3:**
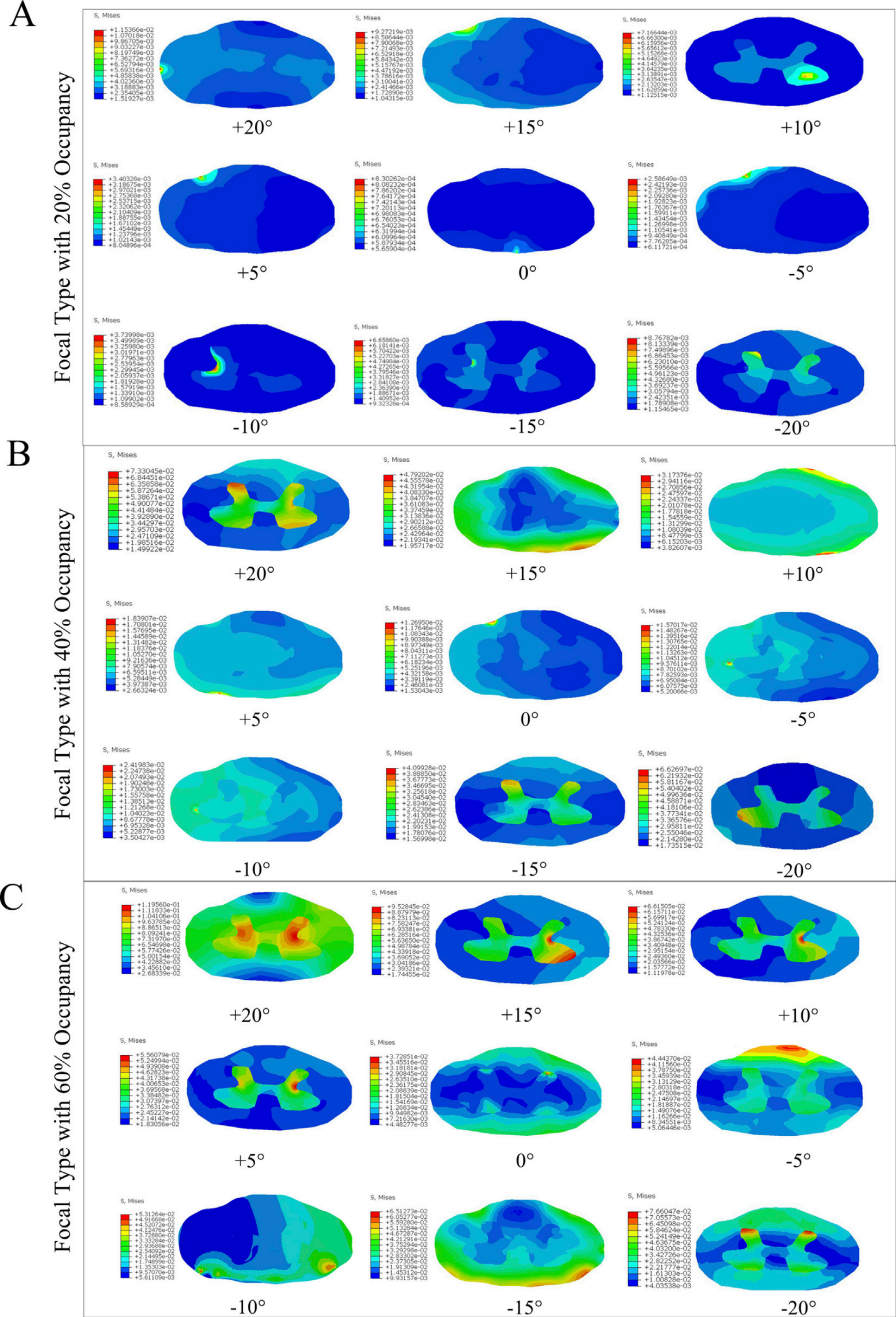
Von Mises stress distribution contours in the spinal cord gray and white matter under different spinal canal occupancy rates and flexion-extension angles for focal C-OPLL. **(A)** Occupancy rate 20%; **(B)** Occupancy rate 40%; **(C)** Occupancy rate 60%. Angles in sequence: extension 20°, 15°, 10°, 5°, neutral 0°, flexion 5°, 10°, 15°, 20°. Stress scale unit: MPa.

**FIGURE 4:**
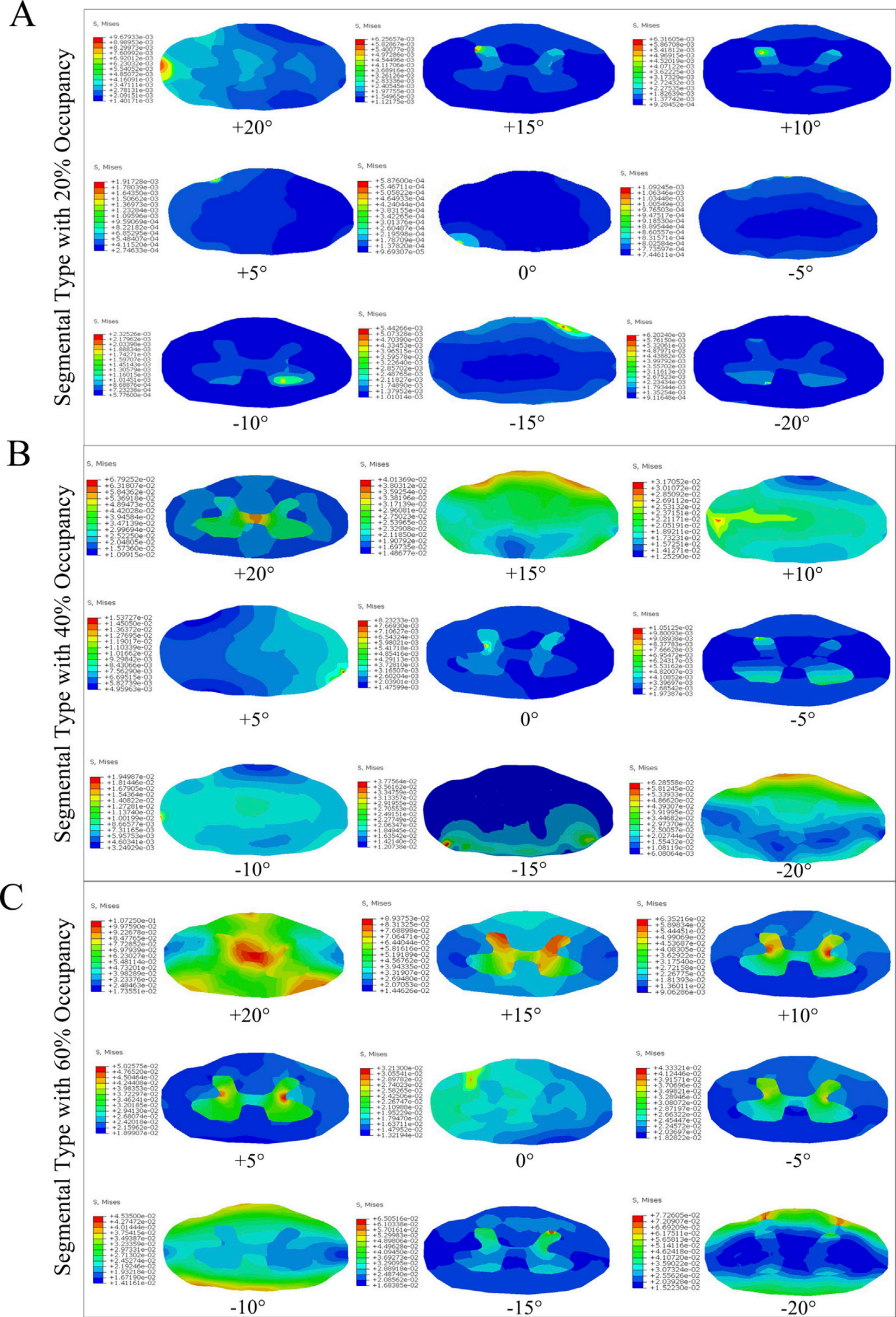
Von Mises stress distribution contours in the spinal dura mater under different spinal canal occupancy rates and flexion-extension angles for focal C-OPLL. **(A)** Occupancy rate 20%; **(B)** Occupancy rate 40%; **(C)** Occupancy rate 60%. Angles in sequence: extension 20°, 15°, 10°, 5°, neutral 0°, flexion 5°, 10°, 15°, 20°. Stress scale unit: MPa.

**FIGURE 5:**
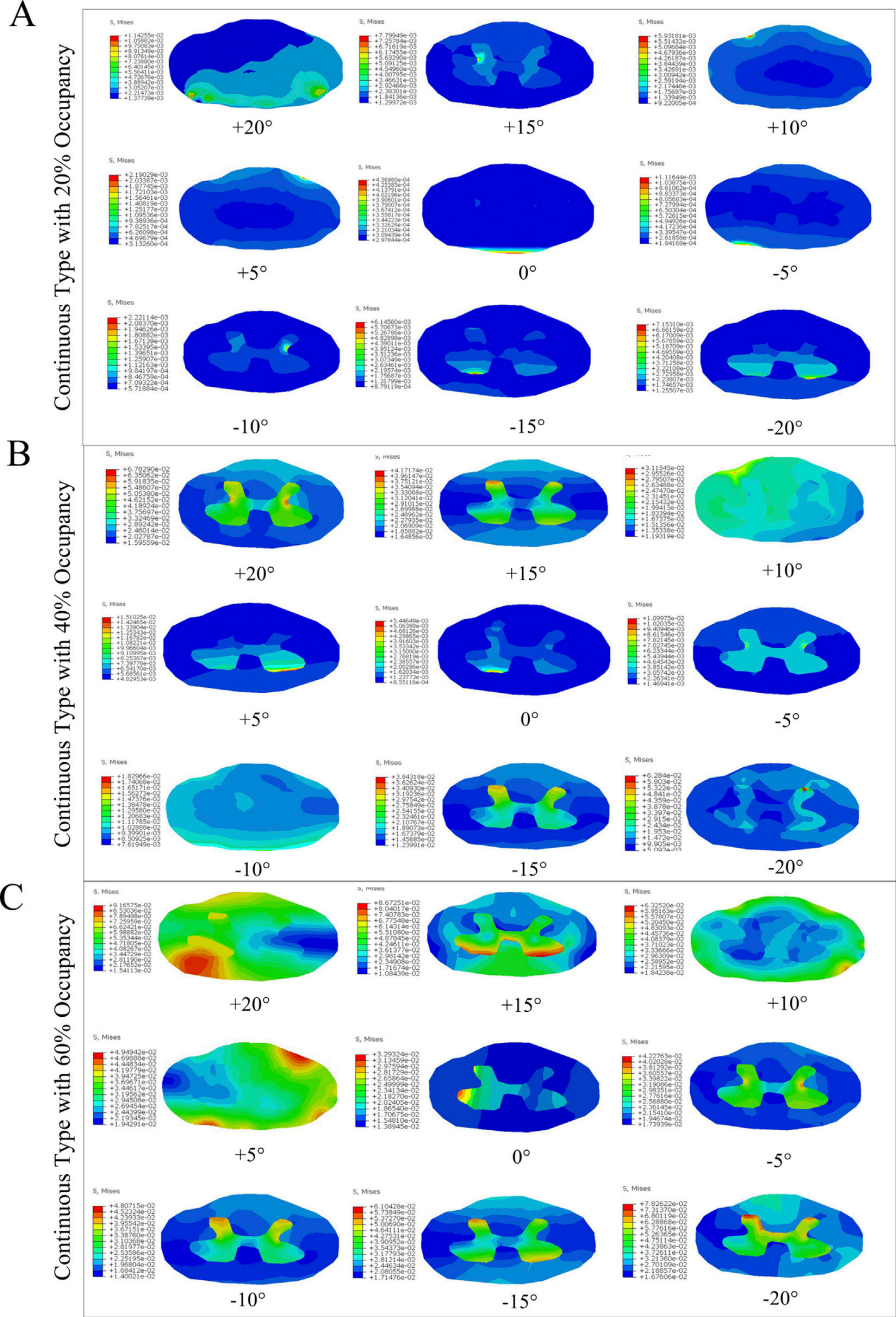
Von Mises stress distribution contours in the spinal cord gray and white matter under different spinal canal occupancy rates and flexion-extension angles for segmental C-OPLL. **(A)** Occupancy rate 20%; **(B)** Occupancy rate 40%; **(C)** Occupancy rate 60%. Angles in sequence: extension 20°, 15°, 10°, 5°, neutral 0°, flexion 5°, 10°, 15°, 20°. Stress scale unit: MPa.

**FIGURE 6:**
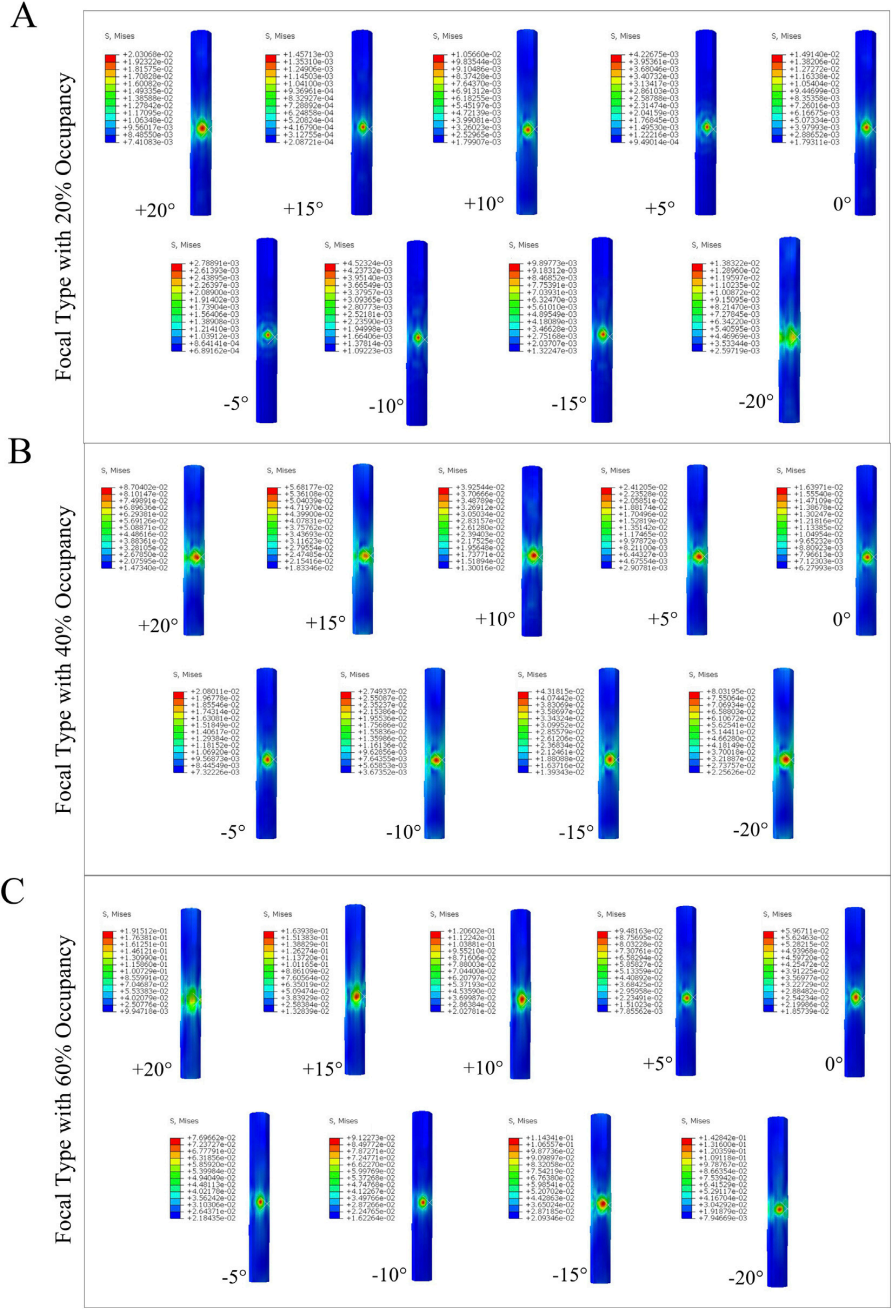
Von Mises stress distribution contours in the spinal dura mater under different spinal canal occupancy rates and flexion-extension angles for segmental C-OPLL. **(A)** Occupancy rate 20%; **(B)** Occupancy rate 40%; **(C)** Occupancy rate 60%. Angles in sequence: extension 20°, 15°, 10°, 5°, neutral 0°, flexion 5°, 10°, 15°, 20°. Stress scale unit: MPa.

**FIGURE 7:**
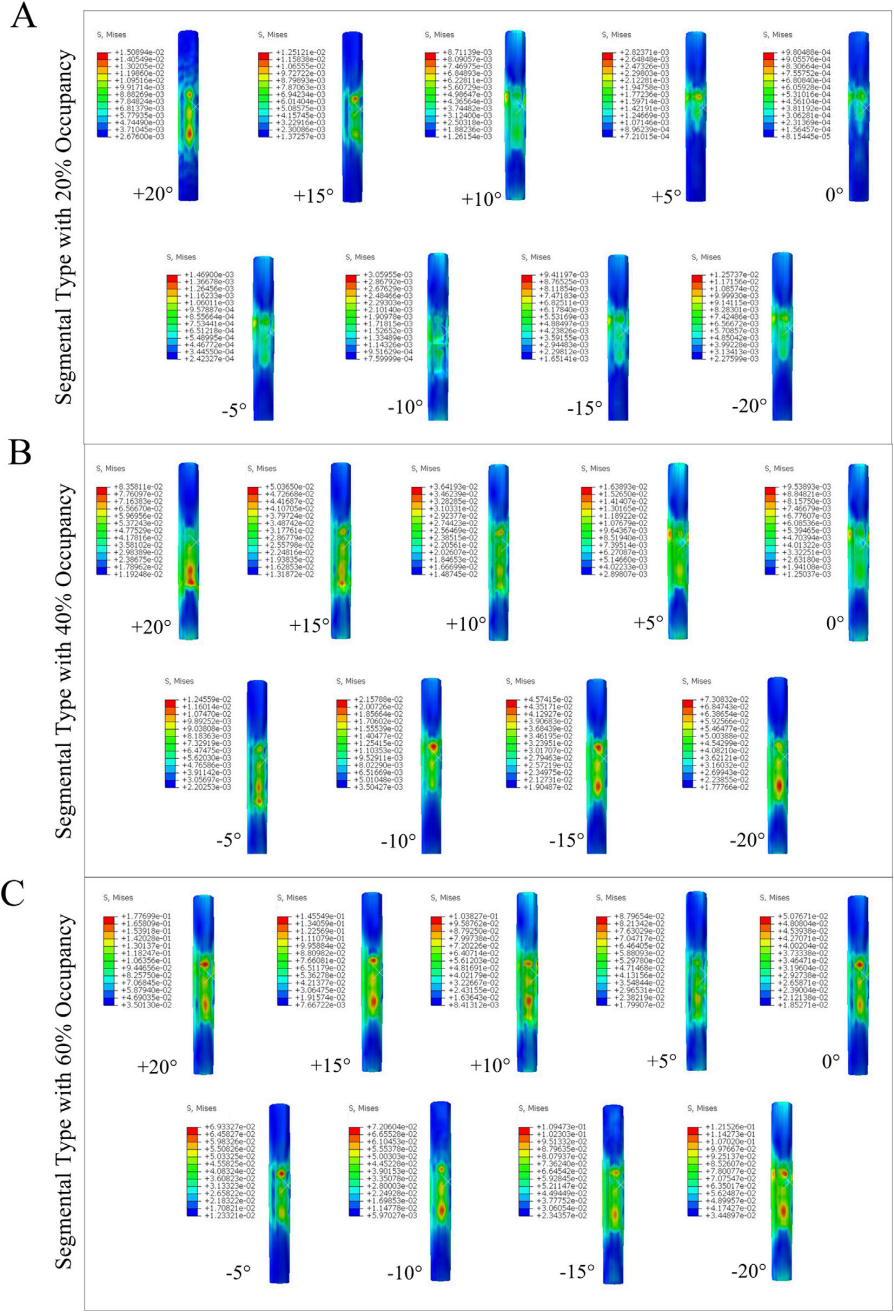
Von Mises stress distribution contours in the spinal cord gray and white matter under different spinal canal occupancy rates and flexion-extension angles for continuous C-OPLL. **(A)** Occupancy rate 20%; **(B)** Occupancy rate 40%; **(C)** Occupancy rate 60%. Angles in sequence: extension 20°, 15°, 10°, 5°, neutral 0°, flexion 5°, 10°, 15°, 20°. Stress scale unit: MPa.

**FIGURE 8:**
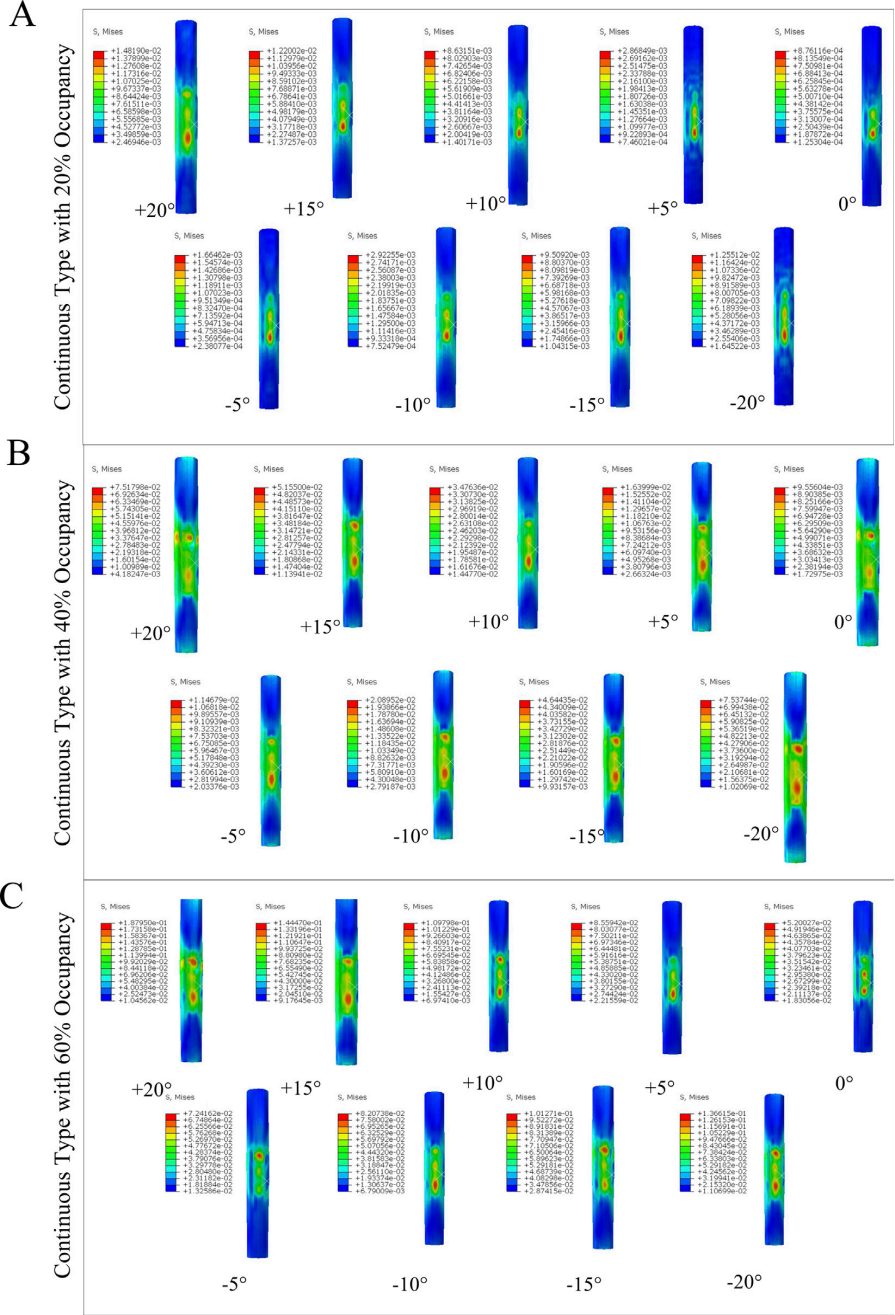
Von Mises stress distribution contours in the spinal dura mater under different spinal canal occupancy rates and flexion-extension angles for continuous C-OPLL. **(A)** Occupancy rate 20%; **(B)** Occupancy rate 40%; **(C)** Occupancy rate 60%. Angles in sequence: extension 20°, 15°, 10°, 5°, neutral 0°, flexion 5°, 10°, 15°, 20°. Stress scale unit: MPa.

Figures 3–8 show the stress contours of the spinal cord gray and white matter and dura mater for the three C-OPLL models under different spinal canal occupancy rates and flexion-extension angles. The stress distribution showed significant differences among the three ossification types. Focal ossification produced pronounced localized stress concentration phenomena under all conditions, with stress contours showing “hot spot” regions, suggesting a “cutting” or “clamping” effect on the spinal cord. The stress distribution in continuous ossification was relatively uniform, extending in a strip-like pattern along the long axis of the ossification. The stress distribution in segmental ossification was intermediate between the two, with stress concentration areas formed at the two independent ossification sites, but the concentration intensity was lower than that of the focal type. As the spinal canal occupancy rate increased, the color of the stress contours for all three models deepened significantly, indicating an overall increase in stress levels; as the flexion-extension angle increased, the stress concentration areas shifted toward the compression center and expanded in scope.

### 3.3 Effect of spinal canal occupancy rate on spinal cord stress

**FIGURE 9:**
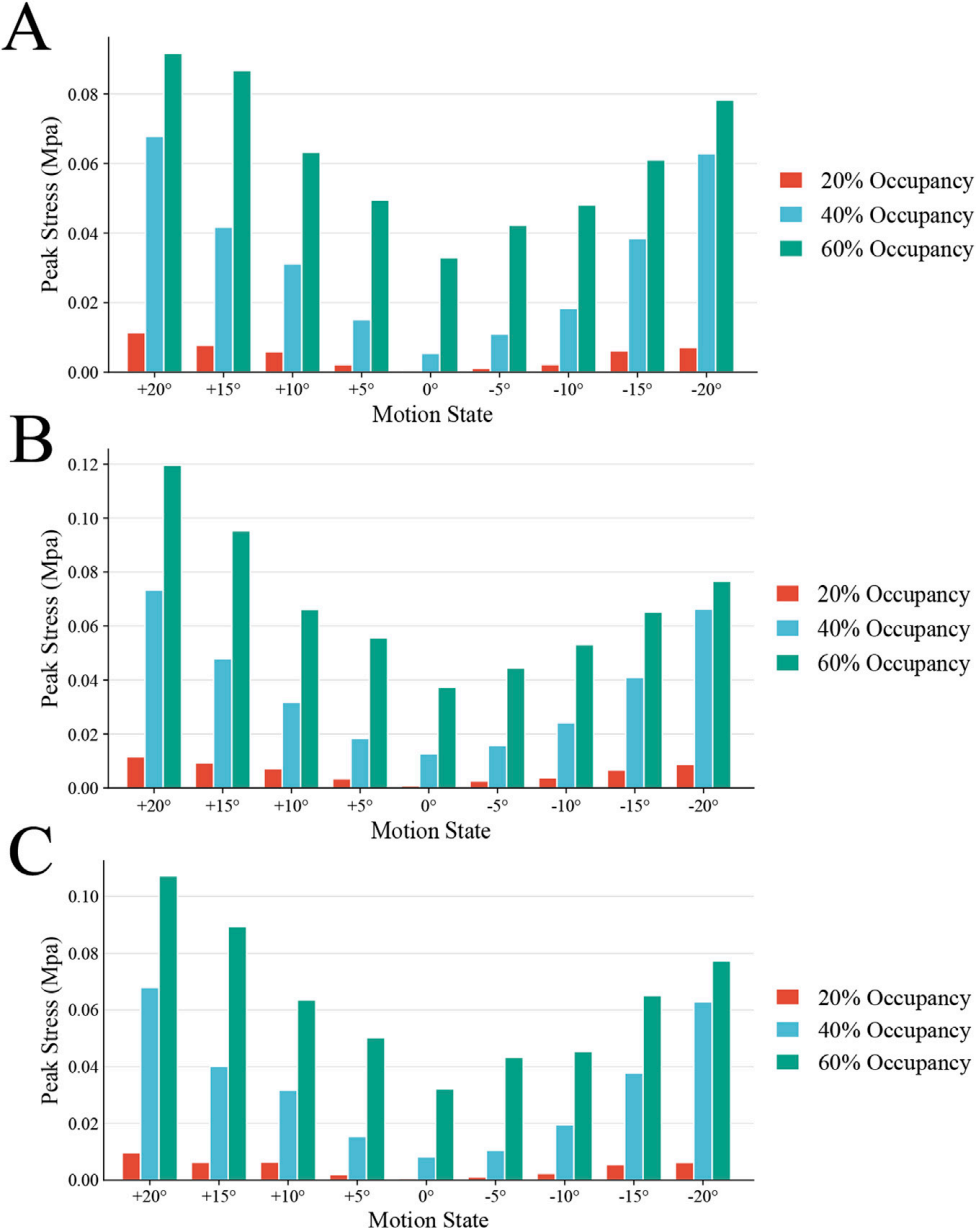
Comparison of peak von Mises stress in the spinal cord gray and white matter among three C-OPLL models under different spinal canal occupancy rates. **(A)** Continuous C-OPLL; **(B)** Focal C-OPLL; **(C)** Segmental C-OPLL. Bar charts display peak stress values across nine flexion-extension angles from 20° flexion to 20° extension.

Figures 9A–C and Supplementary Table S1 show the comparison of peak spinal cord gray and white matter stress for focal, segmental, and continuous C-OPLL under different spinal canal occupancy rates. For continuous C-OPLL, the gray and white matter stresses at neutral position corresponding to 20%, 40%, and 60% occupancy rates were 0.0004°MPa, 0.0054°MPa, and 0.0329°MPa, respectively. From 20% to 40%, stress increased by 12.5 times; from 40% to 60%, stress increased by 6.1 times, with the absolute increment (0.0275 MPa) significantly higher than that of the previous stage (0.0050 MPa). Focal and segmental types showed the same pattern. Notably, focal ossification produced gray and white matter stress of 0.1196 MPa at 60% occupancy rate and maximum flexion, significantly higher than the 0.0917 MPa of continuous type (30.4% higher), suggesting a higher risk of mechanical damage to the spinal cord parenchyma.

### 3.4 Effect of spinal canal occupancy rate on dural stress

**FIGURE 10:**
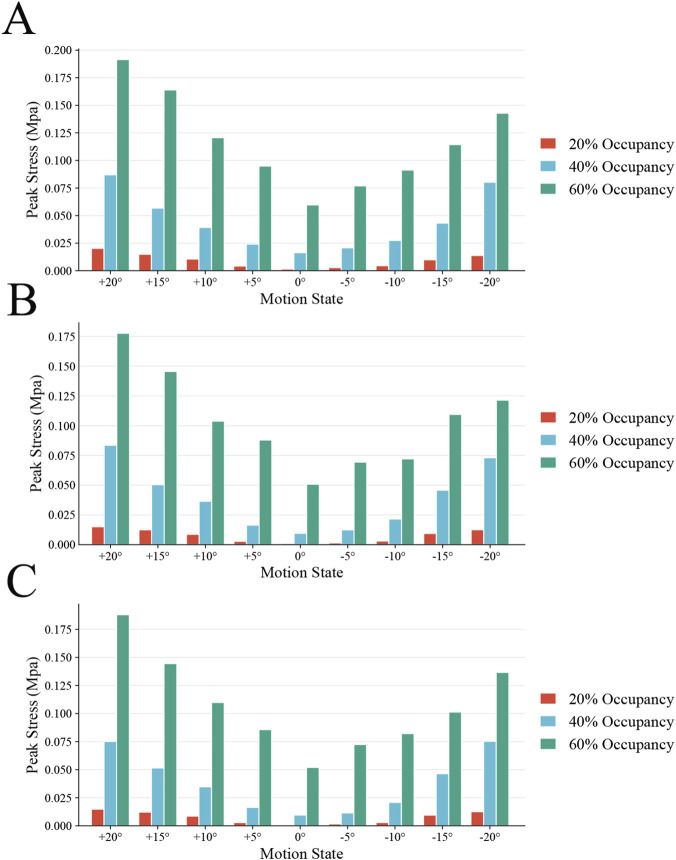
Comparison of peak von Mises stress in the spinal dura mater among three C-OPLL models under different spinal canal occupancy rates. **(A)** Continuous C-OPLL; **(B)** Focal C-OPLL; **(C)** Segmental C-OPLL. Bar charts display peak stress values across nine flexion-extension angles from 20° flexion to 20° extension.

Figures 10A–C and Supplementary Table S2 show the comparison of peak dural stress for focal, segmental, and continuous C-OPLL under different spinal canal occupancy rates. For continuous C-OPLL, dural stress showed nonlinear growth with increasing spinal canal occupancy rate. Taking the neutral position (0°) as an example, the stress peaks corresponding to 20%, 40%, and 60% occupancy rates were 0.0009°MPa, 0.0096°MPa, and 0.0520°MPa, respectively. From 20% to 40%, stress increased by 10.7 times; from 40% to 60%, stress increased by 5.4 times, but the absolute increment (0.0424 MPa) was much higher than that of the previous stage (0.0087 MPa), suggesting that 60% occupancy rate is a key inflection point for sharp stress increase. At maximum flexion (+20°), the stresses corresponding to 20%, 40%, and 60% occupancy rates were 0.0148°MPa, 0.0752°MPa, and 0.1880°MPa, respectively, showing a similar nonlinear growth pattern. Focal C-OPLL and segmental C-OPLL also showed the same trend. Taking focal type at neutral position as an example, the stresses corresponding to 20%, 40%, and 60% occupancy rates were 0.0015°MPa, 0.0164°MPa, and 0.0597°MPa, respectively; from 40% to 60%, the absolute stress increment (0.0433 MPa) was significantly higher than that of the previous stage (0.0149 MPa). This exponential growth pattern persisted across all ossification types and flexion-extension angles, confirming that 60% spinal canal occupancy rate is a universal critical threshold for spinal cord biomechanical tolerance.

### 3.5 Effect of ossification type on spinal cord stress

**FIGURE 11:**
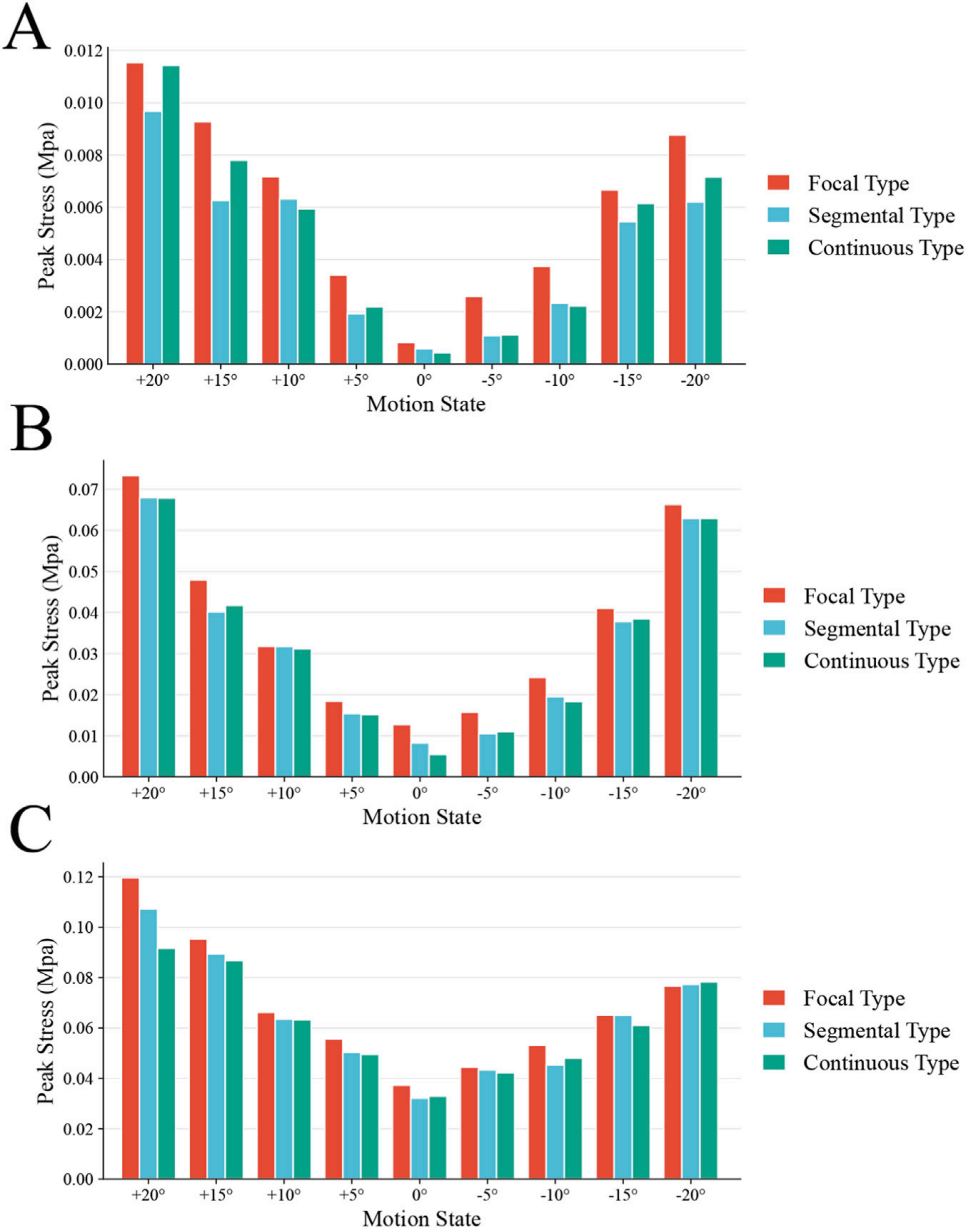
Comparison of peak von Mises stress in the spinal cord gray and white matter among three C-OPLL ossification types under different spinal canal occupancy rates. **(A)** 20% occupancy rate; **(B)** 40% occupancy rate; **(C)** 60% occupancy rate. Line charts illustrate stress variation across the full range of flexion-extension angles from 20° extension to 20° flexion.

Figures 11A–C and Supplementary Table S3 show the comparison of peak spinal cord stress among the three ossification types at 20%, 40%, and 60% spinal canal occupancy rates. The differences among types were more significant for spinal cord gray and white matter than for the dura mater. At 60% occupancy rate and maximum flexion (+20°), the gray and white matter stresses for focal, segmental, and continuous types were 0.1196°MPa, 0.1073°MPa, and 0.0917°MPa, respectively, with focal type 30.4% higher than continuous type. At maximum extension (−20°), the gray and white matter stresses for focal, segmental, and continuous types were 0.0766°MPa, 0.0773°MPa, and 0.0783°MPa, respectively, with continuous type slightly higher than focal type. However, across the full range of flexion-extension angles, focal ossification produced higher gray and white matter stress than continuous and segmental types under most conditions (+20° to −15°), only slightly lower than continuous type at extreme extension (−20°). This phenomenon may be related to the posterior drift of the spinal cord during extreme extension, altering the contact geometry between focal ossification and the spinal cord. Nevertheless, focal ossification produced the highest gray and white matter stress under critical flexion and neutral conditions, suggesting that its overall risk of mechanical damage to the spinal cord parenchyma is higher than that of the other types.

### 3.6 Effect of ossification type on dural stress

**FIGURE 12:**
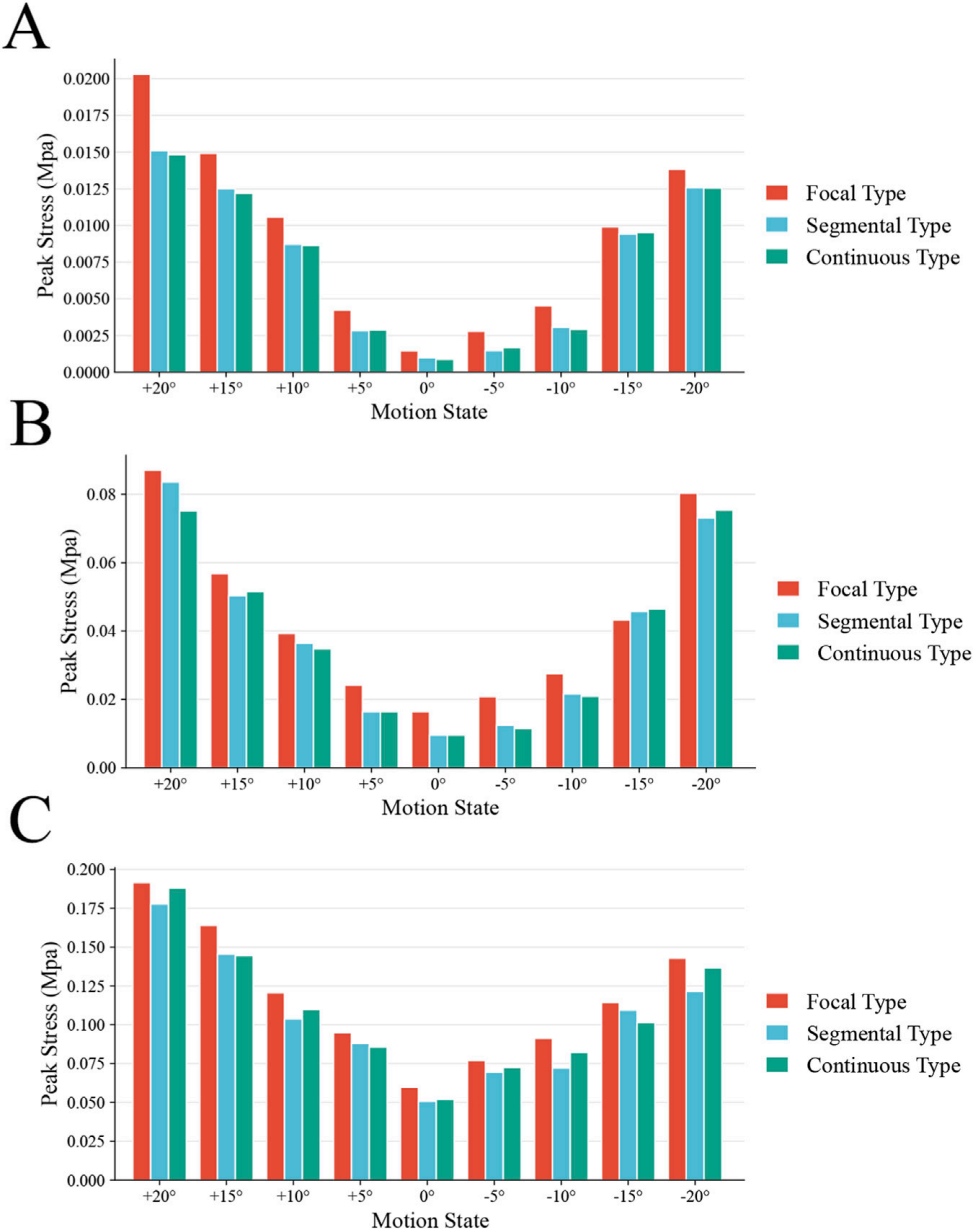
Comparison of peak von Mises stress in the spinal dura mater among three C-OPLL ossification types under different spinal canal occupancy rates. **(A)** 20% occupancy rate; **(B)** 40% occupancy rate; **(C)** 60% occupancy rate. Line charts illustrate stress variation across the full range of flexion-extension angles from 20° extension to 20° flexion.

Figures 12A–C and Supplementary Table S4 show the comparison of peak dural stress among the three ossification types at 20%, 40%, and 60% spinal canal occupancy rates. At 20% occupancy rate, focal ossification produced higher dural stress than continuous and segmental types across all flexion-extension angles. At maximum flexion (+20°), the stresses for focal, segmental, and continuous types were 0.0203°MPa, 0.0151°MPa, and 0.0148°MPa, respectively, with focal type 34.4% higher than segmental type and 37.2% higher than continuous type. At maximum extension (−20°), focal type (0.0138 MPa) was also significantly higher than segmental type (0.0126 MPa) and continuous type (0.0126 MPa). At 40% occupancy rate, this difference became more pronounced. At maximum flexion, the stresses for focal, segmental, and continuous types were 0.0870°MPa, 0.0836°MPa, and 0.0752°MPa, respectively, with focal type 15.7% higher than continuous type. At neutral position, focal type (0.0164 MPa) was 70.8% higher than continuous type (0.0096 MPa), a particularly prominent difference. At 60% occupancy rate, the high risk of focal ossification was further highlighted. At maximum flexion, the stresses for focal, continuous, and segmental types were 0.1915°MPa, 0.1880°MPa, and 0.1777°MPa, respectively, with focal type 7.8% higher than segmental type. At 20° extension, focal type (0.1428 MPa) was also significantly higher than segmental type (0.1215 MPa) and continuous type (0.1366 MPa).

### 3.7 Effect of flexion-extension angle on spinal cord and dural stress

Under all conditions, spinal cord stress showed a monotonically increasing trend with increasing flexion-extension angle, but the rate of increase differed significantly. Under flexion conditions, the stress increase rate was generally higher than under extension, possibly related to the anterior drift of the spinal cord within the spinal canal, resulting in closer contact with the ossification. In continuous type at 60% occupancy rate, dural stress at 20° flexion was 3.6 times that at neutral position (0.1880 vs. 0.0520 MPa), while at 20° extension it was 2.6 times (0.1366 vs. 0.0520 MPa). More importantly, the spinal canal occupancy rate significantly affected the sensitivity of the spinal cord to flexion-extension motion. At 20% and 40% occupancy rates, dural stress showed significant increases at 10° flexion and 15° extension (>200% increase compared to neutral), while changes within ±5° were relatively flat (<50%). However, at 60% occupancy rate, the stress sensitivity threshold decreased significantly: significant stress increases (>60% increase compared to neutral) occurred at 5° flexion and 5° extension, with a continuous upward trend as the angle increased, and no plateau was observed. This finding indicates that under high occupancy rate conditions, the compensatory space of the spinal cord is extremely limited, and even minor cervical spine motion can lead to significant mechanical stress increases.

## 4 Discussion

This study, through high-precision finite element modeling, systematically elucidated the synergistic effects of C-OPLL ossification type, spinal canal occupancy rate, and cervical spine range of motion on spinal cord stress. This is the first study to systematically compare the stress responses of three ossification types under continuous gradients of flexion-extension angles within the same model. The main findings are that focal ossification produced the highest spinal cord stress under all conditions, exhibiting a significant localized concentration effect. Furthermore, 60% spinal canal occupancy rate is the critical threshold for spinal cord biomechanical tolerance; beyond this threshold, stress increased exponentially and dynamic sensitivity increased significantly. Additionally, spinal cord stress was positively correlated with flexion-extension angle, with flexion posing higher risk than extension, and under high occupancy rate conditions, even minor motion (±5°) led to significant stress increases. These findings provide quantitative biomechanical evidence for individualized diagnosis and treatment of C-OPLL.

This study found that focal C-OPLL produced higher spinal cord stress than continuous and segmental types at all occupancy rates and flexion-extension angles, a phenomenon that can be explained by the “stress concentration effect.” According to contact mechanics theory, when a rigid indenter acts on soft tissue, the contact stress is inversely proportional to the curvature radius of the indenter. Focal ossification presents as a localized protrusion, with a smaller contact area with the spinal cord but a smaller local curvature radius, leading to highly concentrated stress at the contact interface. In contrast, continuous ossification extends in a strip-like pattern, with a large contact area and large curvature radius, resulting in relatively uniform stress distribution; although segmental ossification has two compression points, the geometric characteristics of each point are still flatter than those of focal type. This finding is partially consistent with the finite element study by Zheng et al., which also found that ossification type significantly affects spinal cord stress distribution, although that study reported higher stress in segmental OPLL (Zheng et al., 2023). This difference may stem from variations in model construction: Zheng et al. used an ossification model located at multiple segments C4-C6, while this study focused on a single segment C4-C5, suggesting that the location and extent of ossification may affect stress distribution patterns. Biomechanical analysis by Nishida et al. also confirmed that the superposition effect of static compression and dynamic factors varies with ossification morphology (Nishida et al., 2015a). From a pathophysiological perspective, localized stress concentration can lead to axonal damage, demyelination, and microvascular occlusion, triggering irreversible neurological functional impairment (Thapa et al., 2021). The results of this study suggest that focal ossification is a “mechanical high-risk point” in C-OPLL and should be given high clinical attention.

Importantly, these observed differences are deterministic rather than statistical. According to Hertzian contact theory, peak contact stress is inversely proportional to the radius of curvature of the indenter (Johnson, 1985). Focal OPLL, with its small curvature radius, produces pronounced stress concentration; continuous OPLL distributes stress more evenly. The 30.4% difference at 60% occupancy substantially exceeds mesh discretization error (<5%) and expected material parameter uncertainty (±10%–15%), confirming the hierarchy is robust (Ayturk and Puttlitz, 2011; Galle et al., 2010). However, in the absence of established tissue injury thresholds for chronic compression, these stress values should not be directly interpreted as clinical injury risk. For reference, *ex vivo* guinea pig white matter injury occurs at ≈2 kPa and traumatic injury at ≈0.13 MPa (Maikos et al., 2008); our peak stress (0.1196 MPa) falls within this range but requires cautious translation. Thus, the primary value of our findings lies in comparative biomechanics, specifically the relative hierarchy and the 60% threshold, rather than absolute injury prediction. Furthermore, mechanical compression directly induces myelin retraction at the nodes of Ranvier and exposes voltage-gated potassium channels, leading to conduction block; finite element modeling has shown that von Mises stress concentrates at the axoglial interface, where myelin detachment initiates (Ouyang et al., 2010). Animal models of chronic spinal cord compression have consistently demonstrated demyelination, axial loss, and neuronal apoptosis with severity proportional to compression magnitude (Kim et al., 2004). Clinically, the duration and degree of compression correlate negatively with prognosis (Aljuboori and Boakye, 2019). These lines of evidence collectively support that the stress differences identified in this study are biomechanically relevant to the pathogenesis of myelopathy. This is particularly true for the 30.4% higher peak stress in focal OPLL and the exponential increase beyond 60% occupancy. However, the quantitative translation of computed stress values to individual patient outcomes requires further validation through prospective clinical studies.

The results of this study showed that spinal cord stress increased exponentially with increasing occupancy rate, with a significant inflection point at 60%. This phenomenon can be explained by the “compensatory space depletion theory.” Under normal physiological conditions, there is ample cerebrospinal fluid space around the spinal cord, providing buffering space during cervical spine motion and dispersing contact stress (Asif et al., 2025). When the occupancy rate is <40%, the cerebrospinal fluid layer thickness can still be maintained above 1.0°mm, effectively buffering the compression of ossification (Hsiung et al., 2024). When the occupancy rate reaches 60%, the cerebrospinal fluid layer almost completely disappears, and the spinal cord forms a “sandwich-like” entrapment between the ossification and the spinal canal wall, where any additional dynamic displacement directly leads to sharp stress increases (Matsunaga et al., 2002). This finding echoes the clinical study by Ito et al., which found through dynamic CT myelography that as the spinal canal occupancy rate increased, the rate of change in spinal cord cross-sectional area during flexion-extension motion increased significantly, suggesting that the role of dynamic factors is amplified with increasing occupancy rate (Ito et al., 2015a). Fujiyoshi et al. also proposed that the relative importance of static compression and dynamic factors varies with occupancy rate, with dynamic factors dominating neurological deterioration in severe compression (Fujiyoshi et al., 2010). This study further quantified the critical point of this transition – 60% occupancy rate is the overlapping threshold of the static stress inflection point and the dynamic sensitivity turning point (Kim et al., 2013). For patients with occupancy rates ≥60%, even minor cervical spine motions in daily life can lead to sharp increases in spinal cord stress. This mechanism explains why some patients with severe OPLL develop acute spinal cord injury after minor trauma or neck massage.

This study found that spinal cord stress was generally higher during flexion than extension, with a faster rate of increase. This difference may be related to the following anatomical factors: (1) During flexion, the spinal cord drifts anteriorly, increasing the contact area and contact pressure with the ventral ossification (Ito et al., 2013; Joaquim et al., 2019); (2) During flexion, the denticulate ligaments and nerve rootlets are tensioned, generating longitudinal tensile stress that superimposes on the transverse compressive stress (Polak-Kraśna et al., 2019; Wan et al., 2025); (3) During extension, the spinal cord drifts posteriorly but is restricted by the ligamentum flavum and pedicles, resulting in a smaller drift amplitude, and the posterior cerebrospinal fluid layer provides buffering, so the stress increase is relatively moderate (Matsumoto et al., 2024; Zeitoun et al., 2015). This finding complements the study by Xue et al. on cervical rotatory manipulation, which also found that during rotational maneuvers in the flexion position, spinal cord stress was significantly higher than in the neutral and extension positions, suggesting that the flexion position is a high-risk posture (Xue F. et al., 2023). However, unlike this study, Xue et al. reported significant stress increases due to dynamic activity at 40% occupancy rate, which may be related to the rotational activity in their study producing greater shear stress than pure flexion-extension motion. The study by Khuyagbaatar et al. on laminoplasty also confirmed that the degree of postoperative spinal cord posterior shift correlated with preoperative flexion-extension range of motion, suggesting that dynamic factors affect surgical outcomes (Khuyagbaatar et al., 2018). The results of this study suggest that in clinical rehabilitation guidance, restriction of flexion activities should be particularly emphasized, especially in patients with high occupancy rates.

In this study, dural stress was consistently higher than that of the spinal cord gray and white matter, with an average ratio of 1.6–2.1 times. As the first mechanical barrier of the spinal cord, the dura mater plays a key role in buffering external compression (Sakka et al., 2016). Its collagen fiber network can absorb part of the energy, protecting the deep neural tissue (Niestrawska et al., 2023). However, the high stress state of the dura mater also suggests potential damage risk. Long-term high pressure can lead to dural ischemia, fibrosis, and even the formation of intradural adhesions, affecting the normal sliding of the spinal cord (Ren et al., 2023). At 60% occupancy rate and large-angle flexion-extension, dural stress approached 0.2°MPa, approaching the dural injury threshold reported in the literature, suggesting that patients with severe OPLL are at risk of dural damage. This finding is consistent with the study by Xue et al., which also observed that the dura mater bears significantly higher stress than the spinal cord parenchyma under OPLL compression, and that the rate of dural stress increase is faster than that of the spinal cord parenchyma as the occupancy rate increases (Xue F. et al., 2023). Compared to similar studies, Zheng et al. compared stress differences among different OPLL types but focused on stress changes during dynamic flexion-extension without systematically evaluating the angular sensitivity thresholds at different occupancy rates (Zheng et al., 2023). This study, through nine continuous angle gradients, was the first to quantify the stress sensitivity thresholds at different occupancy rates, confirming that 60% occupancy rate is an important node where dynamic sensitivity is significantly enhanced. Secondly, Nishida et al. analyzed the superposition effect of static compression and dynamic factors, but their compression model only set occupancy rates in the 10%–30% range, failing to cover the clinically common severe compression (Nishida et al., 2015a). This study extended the occupancy rate to 60%, revealing the critical threshold for exponential growth. Moreover, this study focused on sagittal flexion-extension motion, which is more consistent with the biomechanical scenarios of daily life activities, and the results can be directly applied to clinical activity guidance. In terms of material modeling, this study used the hyperelastic Mooney-Rivlin model to simulate spinal cord gray and white matter, which can more accurately reflect the nonlinear mechanical behavior of spinal cord tissue compared to the linear elastic models used in previous studies (Galle et al., 2010). The Eulerian-Lagrangian coupling simulation of cerebrospinal fluid also more realistically reproduces fluid-structure interaction effects than traditional fluid cavity models (Rycman et al., 2022).

This study has several limitations. First, the finite element model was constructed based on a single healthy young male volunteer. While this is common in finite element studies, it limits generalizability to all patients. Individual anatomical variations, including spinal canal diameter, spinal cord size, and cervical curvature, may substantially shift actual stress thresholds; thus, the absolute stress values and 60% occupancy threshold reported here should not be used as universal diagnostic cutoffs. Second, the OPLL models were simplified as rigid bodies, which may lead to a minor overestimation (<5%) of peak contact stress but does not affect the relative risk hierarchy. For immature or non-fused OPLL, micromotion could introduce additional dynamic loading not considered here. Third, the study only simulated quasi-static flexion-extension positions, omitting dynamic loading rates, viscoelastic effects, muscle strength, and neural reflexes.

To address these limitations and guide subsequent investigations, future research should focus on: (1) patient-specific models incorporating diverse anatomy (age, sex, cervical curvature, canal diameter) to validate the 60% threshold’s generalizability; (2) prospective clinical cohort studies directly correlating computed stress values with neurological progression; (3) dynamic loading and viscoelastic constitutive models to evaluate rapid motion scenarios (e.g., minor trauma, neck massage); (4) establishment of tissue strain-based failure criteria for chronic compression, enabling a transition from comparative biomechanics to absolute risk prediction. Importantly, the core contribution of the present study lies in the identified nonlinear mechanical principles, namely, exponential stress rise from 40% to 60% occupancy and heightened sensitivity to ±5° motion, and the risk hierarchy (focal > segmental > continuous; flexion > extension). These trends are generalizable, whereas exact inflection points may shift across individuals. Future studies must incorporate patient-specific models and correlate findings with clinical outcomes.

## 5 Conclusion

This single-subject finite element study, with OPLL models placed at C4-C5, shows that focal C-OPLL produces the highest peak spinal cord stress at this level, suggesting a higher mechanical burden. The 60% occupancy rate is a critical biomechanical threshold beyond which stress increases exponentially and becomes highly sensitive to minor motion (±5°). Flexion imposes a greater mechanical burden than extension. Clinically, anterior decompression may be prioritized for focal OPLL, and patients with ≥60% occupancy at C4-C5 should restrict cervical motion, especially flexion. However, because this study is based on a single healthy volunteer, these findings should be considered hypothesis-generating rather than mature clinical conclusions. The relationship between computed stress and clinical injury thresholds, as well as the proposed thresholds, require validation through patient-specific models and prospective multicenter studies.

## Data Availability

The original contributions presented in the study are included in the article/Supplementary Material, further inquiries can be directed to the corresponding author.

## References

[B1] Aljuboori Z. Boakye M. (2019). The natural history of cervical spondylotic myelopathy and ossification of the posterior longitudinal ligament: a review article. Cureus 11, e5074. 10.7759/cureus.5074 31516784 PMC6721920

[B2] Asif H. Visagan R. Boseta E. Zoumprouli A. Papadopoulos M. C. Saadoun S. (2025). Evolution of spinal cord swelling in acute traumatic spinal cord injury. Neurotrauma Rep. 6, 158–170. 10.1089/neur.2025.0005 40129896 PMC11931111

[B3] Ayturk U. M. Puttlitz C. M. (2011). Parametric convergence sensitivity and validation of a finite element model of the human lumbar spine. Comput. Methods Biomech. Biomed. Engin 14, 695–705. 10.1080/10255842.2010.493517 21229413

[B4] Bevill G. Easley S. K. Keaveny T. M. (2007). Side-artifact errors in yield strength and elastic modulus for human trabecular bone and their dependence on bone volume fraction and anatomic site. J. Biomech. 40, 3381–3388. 10.1016/j.jbiomech.2007.05.008 17659290 PMC2099450

[B5] Boruah S. Subit D. L. Paskoff G. R. Shender B. S. Crandall J. R. Salzar R. S. (2017). Influence of bone microstructure on the mechanical properties of skull cortical bone - a combined experimental and computational approach. J. Mech. Behav. Biomed. Mater 65, 688–704. 10.1016/j.jmbbm.2016.09.041 27743944

[B6] Chang H. Kong C. Won H. Kim J. Park J. (2010). Inter- and intra-observer variability of a cervical opll classification using reconstructed ct images. Clin. Orthop. Surg. 2, 8–12. 10.4055/cios.2010.2.1.8 20190995 PMC2824098

[B7] Cui S. Li J. Yu X. Zhao H. Jian F. (2025). Ossification of posterior longitudinal ligament of the cervical spine: a review article. Neurocir. Engl. Ed. 36, 500668. 10.1016/j.neucie.2025.500668 40139271

[B8] Davies B. Schaefer S. Rafati Fard A. Newcombe V. Sutcliffe M. (2024). Finite element analysis for degenerative cervical myelopathy: scoping review of the current findings and design approaches, including recommendations on the choice of material properties. Jmir Biomed. Eng. 9, e48146. 10.2196/48146 38875683 PMC11041437

[B9] Fujiyoshi T. Yamazaki M. Okawa A. Kawabe J. Hayashi K. Endo T. (2010). Static versus dynamic factors for the development of myelopathy in patients with cervical ossification of the posterior longitudinal ligament. J. Clin. Neurosci. 17, 320–324. 10.1016/j.jocn.2009.06.023 20071177

[B10] Galle B. Ouyang H. Shi R. Nauman E. (2010). A transversely isotropic constitutive model of excised Guinea pig spinal cord white matter. J. Biomech. 43, 2839–2843. 10.1016/j.jbiomech.2010.06.014 20832804

[B11] Hsiung W. Lin H. Lin H. Yao Y. Wang S. Chang M. (2024). Mri-based lesion quality score assessing ossification of the posterior longitudinal ligament of the cervical spine. Spine J. 24, 1162–1169. 10.1016/j.spinee.2024.02.007 38365006

[B12] Huang X. Ye L. Wu Z. Liang L. Wang Q. Yu W. (2018). Biomechanical effects of lateral bending position on performing cervical spinal manipulation for cervical disc herniation: a three-dimensional finite element analysis. Evid. Based Complement. Altern. Med. 2018, 2798396. 10.1155/2018/2798396 29991954 PMC6016226

[B13] Hung T. K. Lin H. S. Bunegin L. Albin M. S. (1982). Mechanical and neurological response of cat spinal cord under static loading. Surg. Neurol. 17, 213–217. 10.1016/0090-3019(82)90284-1 7079940

[B14] Ichihara K. Taguchi T. Shimada Y. Sakuramoto I. Kawano S. Kawai S. (2001). Gray matter of the bovine cervical spinal cord is mechanically more rigid and fragile than the white matter. J. Neurotrauma 18, 361–367. 10.1089/08977150151071053 11284555

[B15] Inamasu J. Guiot B. H. Sachs D. C. (2006). Ossification of the posterior longitudinal ligament: an update on its biology, epidemiology, and natural history. Neurosurgery 58, 1027–1039. 10.1227/01.NEU.0000215867.87770.73 16723881

[B16] Ito K. Yukawa Y. Machino M. Kato F. (2013). Spinal cord cross-sectional area during flexion and extension in the patients with cervical ossification of posterior longitudinal ligament. Eur. Spine J. 22, 2564–2568. 10.1007/s00586-013-2982-3 23982905 PMC3886510

[B17] Ito K. Yukawa Y. Ito K. Machino M. Kanbara S. Nakashima H. (2015a). Dynamic changes in the spinal cord cross-sectional area in patients with myelopathy due to cervical ossification of posterior longitudinal ligament. Spine J. 15, 461–466. 10.1016/j.spinee.2014.10.001 25463397

[B18] Ito K. Yukawa Y. Machino M. Kobayakawa A. Kato F. (2015b). Range of motion determined by multidetector-row computed tomography in patients with cervical ossification of the posterior longitudinal ligament. Nagoya J. Med. Sci. 77, 221–228. 25797987 PMC4361524

[B19] Joaquim A. F. Baum G. R. Tan L. A. Riew K. D. (2019). Dynamic cord compression causing cervical myelopathy. Neurospine 16, 448–453. 10.14245/ns.1938020.101 31607076 PMC6790743

[B20] Johnson K. L. (1985). Contact Mechanics. Cambridge: Cambridge University Press.

[B21] Khadka N. Liu X. Zander H. Swami J. Rogers E. Lempka S. F. (2020). Realistic anatomically detailed open-source spinal cord stimulation (Rado-scs) model. J. Neural Eng. 17, 26033. 10.1088/1741-2552/ab8344 32209741

[B22] Khuyagbaatar B. Kim K. Park W. M. Kim Y. H. (2015). Influence of sagittal and axial types of ossification of posterior longitudinal ligament on mechanical stress in cervical spinal cord: a finite element analysis. Clin. Biomech. (Bristol) 30, 1133–1139. 10.1016/j.clinbiomech.2015.08.013 26351002

[B23] Khuyagbaatar B. Kim K. Purevsuren T. Lee S. Kim Y. H. (2018). Biomechanical effects on cervical spinal cord and nerve root following laminoplasty for ossification of the posterior longitudinal ligament in the cervical spine: a comparison between open-door and double-door laminoplasty using finite element analysis. J. Biomech. Eng. 140 (7). 10.1115/1.4039826 29677281

[B24] Kim P. Haisa T. Kawamoto T. Kirino T. Wakai S. (2004). Delayed myelopathy induced by chronic compression in the rat spinal cord. Ann. Neurol. 55, 503–511. 10.1002/ana.20018 15048889

[B25] Kim Y. H. Khuyagbaatar B. Kim K. (2013). Biomechanical effects of spinal cord compression due to ossification of posterior longitudinal ligament and ligamentum flavum: a finite element analysis. Med. Eng. Phys. 35, 1266–1271. 10.1016/j.medengphy.2013.01.006 23419995

[B26] Kong Q. Ma X. Li F. Guo Z. Qi Q. Li W. (2007). Col6a1 polymorphisms associated with ossification of the ligamentum flavum and ossification of the posterior longitudinal ligament. Spine (Phila Pa 1976) 32, 2834–2838. 10.1097/BRS.0b013e31815b761c 18246005

[B27] Kumar R. Kumar A. (2023). Biomechanical analysis of two-level novel cage-type implant for anterior cervical discectomy and fusion: a finite element analysis. J. Long. Term. Eff. Med. Implants 33, 43–52. 10.1615/JLongTermEffMedImplants.2022044668 37522584

[B28] Kwok S. S. S. Cheung J. P. Y. (2020). Surgical decision-making for ossification of the posterior longitudinal ligament versus other types of degenerative cervical myelopathy: anterior versus posterior approaches. Bmc Musculoskelet. Disord. 21, 823. 10.1186/s12891-020-03830-0 33292175 PMC7724709

[B29] Li X. Dai L. (2009). Three-dimensional finite element model of the cervical spinal cord: preliminary results of injury mechanism analysis. Spine (Phila Pa 1976) 34, 1140–1147. 10.1097/BRS.0b013e31819e2af1 19444060

[B30] Liang H. Liu G. Lu S. Chen S. Jiang D. Shi H. (2019). Epidemiology of ossification of the spinal ligaments and associated factors in the Chinese population: a cross-sectional study of 2000 consecutive individuals. Bmc Musculoskelet. Disord. 20, 253. 10.1186/s12891-019-2569-1 31128588 PMC6534908

[B31] Maikos J. T. Qian Z. Metaxas D. Shreiber D. I. (2008). Finite element analysis of spinal cord injury in the rat. J. Neurotrauma 25, 795–816. 10.1089/neu.2007.0423 18627257

[B32] Matsumoto S. Aoyama R. Yamane J. Ninomiya K. Takahashi Y. Kitamura K. (2024). Dynamic cervical spinal canal stenosis: identifying imaging risk factors in extended positions. Asian Spine J. 18, 227–235. 10.31616/asj.2023.0262 38650094 PMC11065511

[B33] Matsunaga S. Kukita M. Hayashi K. Shinkura R. Koriyama C. Sakou T. (2002). Pathogenesis of myelopathy in patients with ossification of the posterior longitudinal ligament. J. Neurosurg. 96, 168–172. 10.3171/spi.2002.96.2.0168 12450279

[B34] Niestrawska J. A. Rodewald M. Schultz C. Quansah E. Meyer-Zedler T. Schmitt M. (2023). Morpho-mechanical mapping of human dura mater microstructure. Acta Biomater. 170, 86–96. 10.1016/j.actbio.2023.08.024 37598794

[B35] Nishida N. Kanchiku T. Kato Y. Imajo Y. Yoshida Y. Kawano S. (2014). Biomechanical analysis of cervical myelopathy due to ossification of the posterior longitudinal ligament: effects of posterior decompression and kyphosis following decompression. Exp. Ther. Med. 7, 1095–1099. 10.3892/etm.2014.1557 24940393 PMC3991514

[B36] Nishida N. Kanchiku T. Kato Y. Imajo Y. Yoshida Y. Kawano S. (2015a). Cervical ossification of the posterior longitudinal ligament: biomechanical analysis of the influence of static and dynamic factors. J. Spinal Cord. Med. 38, 593–598. 10.1179/2045772314Y.0000000221 24964955 PMC4535801

[B37] Nishida N. Kanchiku T. Ohgi J. Ichihara K. Chen X. Taguchi T. (2015b). Mechanical properties of nerve roots and rami radiculares isolated from fresh pig spinal cords. Neural Regen. Res. 10, 1869–1873. 10.4103/1673-5374.170319 26807127 PMC4705804

[B38] Oh C. Noh C. Lee J. Lee S. Hong B. Ko Y. (2021). Diagnostic accuracy of magnetic resonance imaging for sagittal cervical spine alignment: a retrospective cohort study. Int. J. Environ. Res. Public Health 18, 13033. 10.3390/ijerph182413033 34948643 PMC8702200

[B39] Ouyang H. Sun W. Fu Y. Li J. Cheng J. Nauman E. (2010). Compression induces acute demyelination and potassium channel exposure in spinal cord. J. Neurotrauma 27, 1109–1120. 10.1089/neu.2010.1271 20373847 PMC2943505

[B40] Persson C. Evans S. Marsh R. Summers J. L. Hall R. M. (2010). Poisson's ratio and strain rate dependency of the constitutive behavior of spinal dura mater. Ann. Biomed. Eng. 38, 975–983. 10.1007/s10439-010-9924-6 20087767

[B41] Polak-Kraśna K. Robak-Nawrocka S. Szotek S. Czyż M. Gheek D. Pezowicz C. (2019). The denticulate ligament - tensile characterisation and finite element micro-scale model of the structure stabilising spinal cord. J. Mech. Behav. Biomed. Mater. 91, 10–17. 10.1016/j.jmbbm.2018.11.017 30529981

[B42] Ren Z. Xu J. Cheng X. Xu G. Long H. (2023). Pathophysiological mechanisms of chronic compressive spinal cord injury due to vascular events. Neural Regen. Res. 18, 790–796. 10.4103/1673-5374.353485 36204839 PMC9700100

[B43] Rycman A. McLachlin S. Cronin D. S. (2022). Comparison of numerical methods for cerebrospinal fluid representation and fluid-structure interaction during transverse impact of a finite element spinal cord model. Int. J. Numer. Method Biomed. Eng. 38, e3570. 10.1002/cnm.3570 34997836

[B44] Sakka L. Gabrillargues J. Coll G. (2016). Anatomy of the spinal meninges. Oper. Neurosurg. Hagerst. 12, 168–188. 10.1227/NEU.0000000000001048 29506096

[B45] Singh M. Kuharski M. Balmaceno-Criss M. Diebo B. G. Daniels A. (2024). Hyperlipidemia, obesity, and diabetes, and risk of ossification of the posterior longitudinal ligament. World Neurosurg. 188, e642–e647. 10.1016/j.wneu.2024.06.022 38857872

[B46] Stapleton C. J. Pham M. H. Attenello F. J. Hsieh P. C. (2011). Ossification of the posterior longitudinal ligament: genetics and pathophysiology. Neurosurg. Focus 30, E6. 10.3171/2010.12.FOCUS10271 21434822

[B47] Stoner K. E. Abode-Iyamah K. O. Magnotta V. A. Howard M. A. Grosland N. M. (2019). Measurement of *in vivo* spinal cord displacement and strain fields of healthy and myelopathic cervical spinal cord. J. Neurosurg. Spine 31, 53–59. 10.3171/2018.12.SPINE18989 30901756

[B48] Tadepalli S. C. Erdemir A. Cavanagh P. R. (2011). Comparison of hexahedral and tetrahedral elements in finite element analysis of the foot and footwear. J. Biomech. 44, 2337–2343. 10.1016/j.jbiomech.2011.05.006 21742332 PMC7458432

[B49] Teare-Ketter A. LaForme Fiss A. Ebert J. (2021). The utility of neuromotor retraining to augment manual therapy and vestibular rehabilitation in a patient with post-concussion syndrome: a case report. Int. J. Sports Phys. Ther. 16, 248–258. 10.26603/001c.18823 33604153 PMC7872469

[B50] Thapa K. Khan H. Singh T. G. Kaur A. (2021). Traumatic brain injury: mechanistic insight on pathophysiology and potential therapeutic targets. J. Mol. Neurosci. 71, 1725–1742. 10.1007/s12031-021-01841-7 33956297

[B51] Tsuyama N. (1984). Ossification of the posterior longitudinal ligament of the spine. Clin. Orthop. Relat. Res. 184, 71–84. 10.1097/00003086-198404000-00010 6423334

[B52] Wan C. Shen X. Wu X. Yu C. Shao Y. Zhang R. (2025). Assessing the biomechanics of scheuermann's kyphosis affected thoracolumbar spine in forward flexion at the tissue-level using a finite element model. Sci. Rep. 15, 27408. 10.1038/s41598-025-12968-7 40721849 PMC12304476

[B53] Wang P. Liu X. Zhu B. Ma Y. Yong L. Teng Z. (2018). Association of il17rc and col6a1 genetic polymorphisms with susceptibility to ossification of the thoracic posterior longitudinal ligament in Chinese patients. J. Orthop. Surg. Res. 13, 109. 10.1186/s13018-018-0817-y 29764467 PMC5952594

[B54] Xue F. Deng H. Chen Z. Yang H. Li Y. Yuan S. (2023a). Effects of cervical rotatory manipulation on the cervical spinal cord complex with ossification of the posterior longitudinal ligament in the vertebral canal: a finite element study. Front. Bioeng. Biotechnol. 11, 1095587. 10.3389/fbioe.2023.1095587 36714008 PMC9880201

[B55] Xue H. Tang D. Zhao W. Chen L. Liao Z. Xue J. (2023b). Static mechanical analysis of the vertebral body after modified anterior cervical discectomy and fusion (partial vertebral osteotomy): a finite element model. J. Orthop. Surg. Res. 18, 554. 10.1186/s13018-023-04033-8 37528421 PMC10391851

[B56] Yu J. Manouchehri N. Yamamoto S. Kwon B. K. Oxland T. R. (2020). Mechanical properties of spinal cord grey matter and white matter in confined compression. J. Mech. Behav. Biomed. Mater. 112, 104044. 10.1016/j.jmbbm.2020.104044 32947099

[B57] Zeitoun D. El Hajj F. Sariali E. Catonné Y. Pascal-Moussellard H. (2015). Evaluation of spinal cord compression and hyperintense intramedullary lesions on t2-weighted sequences in patients with cervical spondylotic myelopathy using flexion-extension mri protocol. Spine J. 15, 668–674. 10.1016/j.spinee.2014.12.001 25485484

[B58] Zheng L. Cao Y. Yang Y. Xu M. Zeng H. Zhu S. (2023). Effect of different types of ossification of the posterior longitudinal ligament on the dynamic biomechanical response of the spinal cord: a finite element analysis. J. Biomech. Eng. 145, 121002. 10.1115/1.4063194 37578172

